# Analysis of SEF95 derived from two processed EEG devices during pediatric anesthesia for non-cardiac surgery

**DOI:** 10.1007/s10877-025-01338-3

**Published:** 2025-08-13

**Authors:** Zaccaria Ricci, Lorenzo Gobbi, Enrica La Rosa, Elena Filippini, Matteo Lui, Maximilan Fischer, Denise Colosimo, Stefano Romagnoli

**Affiliations:** 1 Department of Emergency and Critical Care, Anesthesia and Pediatric Intensive Care Unit, Meyer Children’s Hospital, IRCCS, Florence, Italy; 2 Department of Health Science, Section of Anesthesia and Intensive Care, University of Florence, Piazza di San Marco 4, Florence, Italy; 3 Department of Anesthesia and Intensive Care, Azienda Ospedaliero- Universitaria Careggi, Florence, Italy

**Keywords:** Depth of anesthesia, Neuromonitoring, Neurodevelopment, Processed electroencephalography

## Abstract

This study aimed to compare the values of spectral edge frequency at the 95th percentile (SEF95) obtained simultaneously by two different processed electroencephalography monitors (BIS^®^ and SedLine^®^) in pediatric patients of varying ages undergoing non cardiac surgery to determine whether they remain reproducible regardless of the equipment used. Tertiary Pediatric Hospital. We conducted a prospective observational study involving pediatric patients aged 12 months to 18 years. Patients were excluded if the sensor fit was inadequate or if artifacts interfered with the data collection. Anesthesia was administered by anesthesiologists according to their preferences. A total of 51 children were enrolled, yielding 402 paired BIS/SedLine SEF95 values. These values showed an r² of 0.73 at linear regression analysis (*p* < 0.0001), with a bias of 0.62 (2.4) Hz and 95% limits of agreement (LoA) ranging from − 4.08 to 5.32 Hz in Bland-Altman analysis. Median SEF95 deltas (i.e., differences of paired BIS/SedLine SEF95 values) across the analyzed time points showed significant differences (*p* = 0.0017) between values at 15 min and 60 min after skin incision compared to extubation. A delta SEF95 within the ± 2 Hz range was observed in 267 cases (66%), within ± 3 Hz occurred in other 67 measurements (17%) and within ± 4 Hz in further 48 (12%). The remaining 20 measurements showed a higher delta. SedLine SEF95 was higher than BIS in 40 cases, while BIS was higher than SedLine in 96 cases. SEF95 monitored by BIS or SedLine pediatric patients showed some differences, with deltas up to ± 4 Hz. Values appeared to be closer during the anesthesia maintenance phase. The clinical relevance of these findings should be further confirmed.

**Supplementary Information:**

The online version contains supplementary material available at 10.1007/s10877-025-01338-3.

## Introduction

The Spectral Edge Frequency 95 (SEF95) is a quantitative electroencephalography (EEG) parameter defined as the frequency below which a 95% of total EEG power is found in the power spectrum [1]. Power analysis is derived from a raw EEG trace using the Fast Fourier Transform, which isolates individual frequencies during an EEG epoch, quantifies those frequencies (referred to as “power” in spectral analysis), and assesses their phase [1].

SEF95 is a continuous parameter: in an awake patient, it typically ranges from 22 to 25 Hz, a moderately sedated patient exhibits a SEF95 between 14 and 18 Hz, while a SEF95 of 12 Hz during general anesthesia suggests that most electrical activity in a patient’s frontal brain lies below the frequency of the alpha band [2]. When progressing to deeper anesthesia values between 8 and 10 Hz, or lower, can be reached.

Recently, EEG-targeted anesthesia has been proposed for both adult [3] and pediatric patients [4, 5], with some studies reporting significant clinical improvements by guiding anesthetic drug administration based on this approach. However, processed EEG (pEEG) has primarily been applied in these studies. PEEG is a quantitative analysis in which dimensionless-derived anesthesia indices are computed into a single value [1]. Unsurprisingly, these algorithms do not account for age-related physiological changes in the EEG which reaches a fully mature adult pattern by 18–20 years old [6–8]. To date, no pediatric pEEG algorithm has been developed.

Theoretically, SEF could be used regardless of age, as it results from the standardized processing of raw traces rather than from a complex algorithm that must account for various features of the mature brain. Although SEF95 is not a reliable metric to assess the adequacy of anesthesia level if used as a single parameter, it is reasonable to consider that SEF95 can be an additional value to be implemented into pEEG monitoring in children [1]. A recent study demonstrated the feasibility of using SEF95 to guide EEG-based anesthesia in infants aged 3 months to 1 year [9]. Furthermore, it can be considered a quantitative representation of the density spectral analysis, that is increasingly used as pediatric anesthesia guide [10, 11]. Additionally, the SEF is calculated using the same protocol across all devices, making it theoretically applicable at the bedside in centers using different monitors, provided that shared protocols and specific anesthesia depth thresholds were established.

The aim of this study was to compare the SEF95 values obtained simultaneously by two different monitors in pediatric patients undergoing non cardiac surgery of varying ages to determine whether this value is reproducible across different equipment.

## Methods

A prospective observational study was conducted from April to November 2024 at Meyer Children’s Hospital, IRCCS, in Florence, Italy. SedLine^®^ (Masimo, Irvine, CA, USA) and BIS^®^ (Medtronic, Dublin, Ireland) monitors were used. Written informed consent was obtained from parents or legal guardians. Pediatric patients aged over 12 months and under 18 years were included, with no specific exclusion criteria. Cases were excluded after screening if the forehead had not enough space for two sensors or if there was poor sensor fit. Post-enrollment, patients were excluded if artifacts interfered with data collection or if data were missing (at least three time points were required from both monitors). Sensor placement followed the scheme proposed in a previous adult study [12] and is also presented in supplementary Fig. 1. The study was approved by the Hospital Ethics Committee (Comitato Etico Regionale Pediatrico per la Sperimentazione Clinica della Regione Toscana, n. 310/2022). Strengthening the Reporting of Observational Studies in Epidemiology (STROBE) guidelines were applied [13]. SEF95, the percentage of electromyography (EMG) activity, BS rate, and signal quality were collected using both devices. Adult and pediatric sensors were used for both machines according to manufacturer indications. Data were recorded manually by one of the study authors, who was not directly involved in anesthesia. Data collection occurred at induction, during laryngeal mask or endotracheal tube placement, at surgical incision, and every 15 min (min) thereafter until the end of surgery, including extubation and discharge from the operating room. Since SedLine provides bilateral data, only homolateral values were considered (i.e., if the BIS sensor was placed on the right forehead, the right SEF95 from SedLine was analyzed alongside the BIS SEF95).

Demographic data, including diagnosis, American Society of Anesthesia (ASA) score, surgical procedure, and type of anesthesia (i.e., inhalational or total intravenous, general vs. general with locoregional), were also recorded. Anesthesia was administered by anesthesiologists according to their personal preferences, as no strict protocol was followed at our institution. The anesthesiologist could choose to utilize either SedLine or BIS monitoring, or none at all, for anesthesia depth monitoring. Vital parameters (i.e., mean arterial pressure, oxygen saturation, end-tidal CO2, and heart rate) were recorded at the same time points as the EEG monitoring.

The primary aim of our prospective observational study was to determine the median absolute values of all SEF95 measurements from the BIS and SedLine at each time point, as well as the median SEF95 differences (delta SEF95) for each paired measurement at the analyzed time points. Secondary aims included assessing the correlation and bias between the monitors and describing how often the delta SEF95 exceeded ± 2, ±3 or ± 4 Hz (i.e., a difference above or below the reference number: if 10 Hz is the reference, a ± 2 delta implies a value of 8–12 Hz in the comparator). Finally, we aimed to identify the predictors of the analyzed differences.

### Statistical analysis

All data are presented as median (interquartile range). The Mann-Whitney test was used to assess differences between variables. Linear regression (r²) or Pearson correlation (r and 95% confidence interval, CI) were used to verify the association between continuous variables. Logistic regression was applied to dichotomous variables. A one-way analysis of variance, a two-way analysis of variance, and mixed-effects analysis were performed to analyze the differences over time between continuous variables. Tukey’s multiple comparisons post-hoc test was used to assess differences between groups at individual time points. Bland-Altman analysis was applied to compare measurements from the two monitors, reporting bias (standard deviation) and 95% limits of agreement (LoA). A p-value of < 0.05 was considered statistically significant. Statistical analysis was performed using the GraphPad Prism 9.0 software package (GraphPad Software, San Diego, CA, USA).

We aimed to enroll 50 patients as an exploratory sample, ensuring the representation of all predefined age subgroups (i.e., < 2, 2–5, 5–10, and > 10 years) in proportion to routine activity at our hospital. Anticipating a 20% dropout rate and variations in surgery durations, we planned to enroll up to 50 patients to target at least 40 patients being monitored for at least four time points.

## Results

A total of 60 patients were screened. Four patients were excluded due to inadequate forehead space for proper sensor fitting. After enrollment, 5 patients were excluded due to artifacts or missing data that hindered EEG reading (4 due to SedLine and 1 due to the BIS sensor) (supplementary Fig. 2). Table 1 presents the baseline and demographic data of the 51 analyzed children and their age groups. Vital parameters (heart rate and mean arterial pressure) are shown in Supplementary Fig. 3, while oxygen saturation remained stable between 95% and 100%, and end-tidal CO2 levels ranged from 35 to 40 mmHg throughout all procedures for all patients.

**Table 1:** General demographics and baseline data. Absolute numbers (n.) or median (interquartile range). Pts: patients. ASA: American society of anesthesiology. ENT: ear nose and throat. LMA: laryngeal mask airway

Pts *n*.	51
Age (years)	8 (6–10)
Age groups (years: pts n.)	>1 and < 2: 4 2–5: 7 5–10: 28 >10: 12
Weight (Kg)	27.5 (21.4–38)
Surgery duration (min)	107 (68-160.5)
Heart rate (bpm)	93.5 (80.2-112.5)
Mean arterial pressure (mmHg)	73.5 (64-81.8)
ASA (score: pts n.)	1: 22 2: 21 3: 8
Surgery (type: pts n.)	General surgery = 9 Head (ENT and oculist) = 6 Orthopedics: 24 Urology: 8 Other: 4
Surgery (major/minor)	27/24
Comorbidities (yes/no)	19/32
Locoregional anesthesia (yes/no)	35/16
Number of patients enrolled at different time points (time point: pts n.)	Induction: 19
	LMA/intub: 32
	skin incision: 51
	15 min: 49
	30 min: 47
	45 min: 37
	60 min: 30
	75 min: 22
	90 min: 14
	105 min: 12
	120 min: 8
	Extubation: 47
	Discharge: 34

**Fig. 1:**
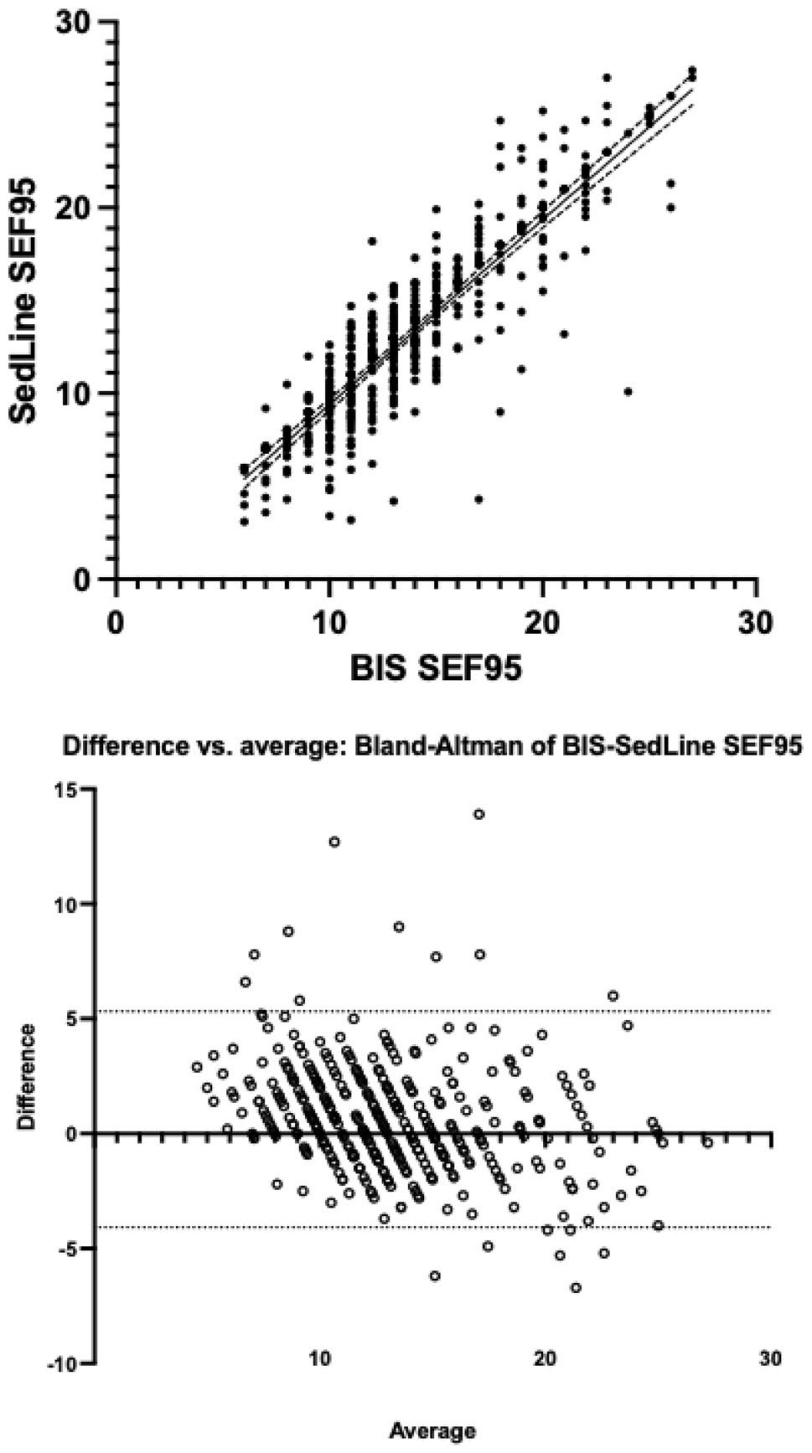
Panel A: linear regression between BIS-SEF95 and SedLine-SEF95 (r² 0.73, *p* < 0.0001); Panel B: Bland Altman analysis (bias of 0.62 (2.4) Hz and 95% limits of agreement from − 4.08 to 5.32 Hz) between BIS-SEF95 and SedLine-SEF95

Overall, at “skin incision”, “15 min”, “30 min” and “extubation” data from 51, 49, 47 patients were collected, respectively, with a loss of 4 patients’ data (Table 1). After the first 30 min the number of enrolled patients decreased due to the end of surgery: at 60 min data from 30 patients were available, and they became 8 at 120 min (Table 1). Finally, a total collection of 406 BIS and 408 SedLine values was available. Of these, 402 paired values were included due to 4 missing BIS values and 2 missing SedLine values. The analysis of SEF95 from the two monitors revealed a significant correlation in linear regression analysis (r² 0.73, *p* < 0.0001) with a bias of 0.62 (2.4) Hz and 95% limits of agreement (LoA) ranging from − 4.08 to 5.32 Hz in the Bland-Altman analysis (Fig. 1A and B). A delta SEF95 within the ± 2 Hz range was observed in 267 cases (66%), within ± 3 Hz occurred in other 67 measurements (17%) and within ± 4 Hz in further 48 (12%). The remaining 20 measurements (5%) showed a higher delta (range − 5 to + 6).

**Fig. 2:**
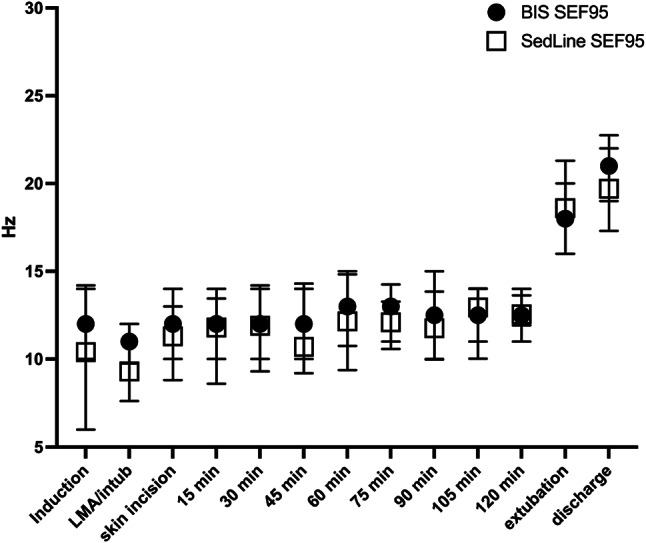
Median (interquartile range) absolute values of BIS-SEF95 and SedLine-SEF95 during general anesthesia for pediatric surgeries. Abbreviations: LMA: laryngeal mask airway

BIS SEF95 and SedLine SEF95 absolute values over time are presented in Fig. 2. Median values over time of the two monitors ranged from 11.30 Hz to 12.88 Hz during the maintenance phase. The lowest values were observed at laryngeal mask/endotracheal tube positioning (i.e., 10.25 Hz) whereas the highest values were observed at extubation and discharge (i.e., 18.2 and 20 Hz, respectively). Median SEF95 absolute values of the two monitors did not show significant differences over time (mixed-effects analysis for repeated measures; device factor *p* = 0.35).

**Fig. 3:**
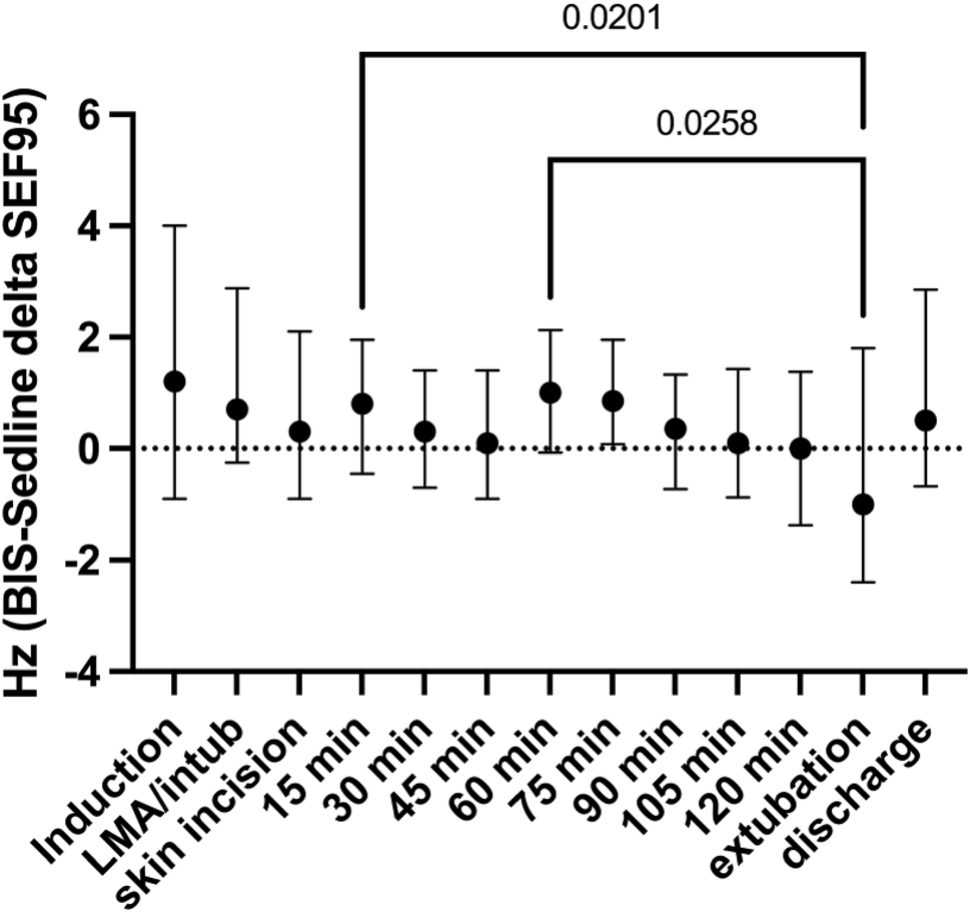
BIS-SedLine delta SEF95 over time (*p* = 0.0017 at one-way ANOVA; *p* = 0.02 and *p* = 0.025 at Tukey’s multiple comparisons post-hoc test between delta SEF95 at extubation vs. 15 min and 60 min, respectively). Abbreviations: LMA: laryngeal mask airway

The analysis of median delta SEF95 over time at one-way ANOVA revealed significant variations (*p* = 0.0017). Specifically, Tukey’s multiple comparison test identified significant delta SEF95 differences between the 15-minute (*p* = 0.02) and 60-minute (*p* = 0.026) time points compared to extubation (Fig. 3). All median delta SEF95 were within 0.3 and 0.9 Hz except at induction (1.2, 0.9-4, Hz), and extubation (−1, −2.4-1.8, Hz).

SedLine SEF95 values were higher than BIS in 40 cases, whereas BIS values exceeded SedLine in 96 cases. No apparent systematic over- or underestimation was observed when individual data point differences were analyzed (data not shown).

When ages’ impact on delta SEF95 was evaluated, older patients showed higher delta SEF95 at extubation (*r* = 0.38, 95% CI 0.10 to 0.60, *p* = 0.0082) and that it had no impact at other time points. Furthermore, overall median delta SEF95 stratified in the predefined age subgroups showed significant differences between patients older than 10 years old and those between 2 and 5 years old (Table 2). The analysis of other predictors of delta SEF95 showed that the left BIS site was associated with greater delta SEF95 at incision (*r* = 0.32, 95% CI 0.04 to 0.54, *p* = 0.02) and at 15 min (*r* = 0.35, 95% CI 0.08 to 0.58, *p* = 0.01). No associations were found with ASA score, mean arterial pressure, surgery duration, type of anesthesia (inhalational vs. intravenous or general vs. general with locoregional), type of surgery (major vs. minor), or percentage of EMG activity.

**Table 2:** Differences of SEF95 values between predefined age groups. Data are expressed as median (interquartile range). *P* = 0.001 at 1way ANOVA and 0.008 at tukey’s multiple comparisons post-hoc test between 2–5 and > 10 years old (*)

Age groups (years)	> 1 and <2	2–5	5–10	> 10
SEF 95 differences	0.5 (−0.95 to 2.15)	0 (−1.27 to 1.35)*	0.4 (−0.9 to 0.4)	0.65 (−0.57 to 2.67)*

## Discussion

The key findings of this study are as follows: (1) SEF95 analyzed by BIS and SedLine monitors showed that these machines cannot be considered interchangeable in 100% of cases. (2) The highest deltas appeared to occur during the “dynamic” phases of anesthesia (the so called “LOC and ROC”, loss of conscience and recovery of conscience): during induction, extubation, and awakening some oscillation in SEF95 values may occur, and rapid changes can be tracked differently by the two devices, especially when data are recorded manually. (3) Conversely, during the maintenance phase of anesthesia, when anesthetic stability is most common, the SEF95 values from the BIS and SedLine were closer. (4) There appeared to be no substantial clinical predictors of delta SEF95, and they seemed rather unpredictable, with BIS more frequently reporting higher values compared to SedLine.

Our data showed that SEF95 measurement may not be considered as a standard value, regardless of the chosen monitoring device. With these findings we cannot suggest that one machine is more precise than the other. It is currently unknown what is the clinical relevance of the differences we found. It must be acknowledged that even if the majority of deltas, mostly in the maintenance phase, were below 2 Hz, a difference that would not trigger any anesthetic modification (i.e., having one monitor saying 8 Hz and the other saying 6–10 Hz), it could be argued that a difference of 4 Hz (or even higher) might imply that one monitor is indicating alpha loss and the other an adequate anesthetic plan (i.e., 16 vs. 12 Hz). The velocity of the data refresh may be an issue. In fact, this aspect is managed very differently by the two monitors: BIS calculates data every 15 s by default (customizable to 10, 15, or 30 s), while SedLine refreshes data every 1–2 s. Consequently, BIS data and trend lines may appear smoother, whereas SedLine tends to display more oscillations (or instantaneous values) that can be more pronounced during deepening or emergence from anesthesia. In this regard, we speculate that since the highest differences were found at “ROC”, the positive correlation of delta SEF95 at extubation with age might be due to the fact that older children show quick EEG modifications at awakening that are captured more precisely by SedLine, contributing to a greatest difference. Ultimately, the interchangeability of the two machines must be argued: if more granular data are deemed appropriate by the operator, SedLine may be the preferred monitor. Conversely, if a stable signal is preferred due to the lack of need for rapid anesthetic adjustments, BIS may provide more comfortable information.

We consider SEF95 a potentially valuable contributor to EEG analysis during pediatric anesthesia as confirmed by several recent studies ([9–11, 14]– [15]). However, regardless of the SEF95 application during pediatric anesthesia, our data described that SEF95 monitoring may be affected by the choice of the monitor type.

### Limitations

This study has several limitations. First, the sample size was selected arbitrarily, as this study was a technical evaluation without previously available data. Second, this study cannot be considered in any way a proof of the adequacy of frontal EEG monitoring and SEF95 application for assessing anesthesia depth in children. However, similar to our previous study [16], the SEF95 data were consistent with the expected anesthesia depth during various surgical phases (i.e., induction, skin incision, maintenance, extubation, and discharge). In our study delta SEF95 appeared to be higher at induction and at extubation. We acknowledge that few data were available at induction because in our center we put the sensor to the awake patient only in collaborative children (who are the minority). Third, we conducted manual data collection to simulate real-world EEG monitoring by anesthesiologists. More granular data downloaded directly from the devices might have allowed for better interpretation of SEF95 oscillations during different anesthetic phases. However, we chose to conduct this explorative observation in a “real world manner” by retrieving the numbers with the eye of the bedside clinician, who glances at the monitor numbers every once in a while. Additionally, we could not assess the role of EMG, which is filtered differently between the two devices; however, it appears that EMG was not associated with delta SEF95. While sensor positioning might have affected the accuracy of the monitoring systems, this primarily occurred with the BIS monitor, as SedLine was positioned in its recommended location. Furthermore, it is important to note that no study has evaluated whether sensor placement affects EEG readings. It is possible that, in real-world applications, the impact of exact sensor position in the forehead is minimal. Our study, like many other evaluations of pediatric EEG for anesthesia depth monitoring ([4]– [5, 9, 14]), predominantly included low ASA, older children. We cannot exclude the possibility that, in more complex surgeries (e.g., pediatric cardiopulmonary bypass) and/or in younger patients (i.e., children < 12 months old), significant impacts on SEF95 readings may exist, potentially affecting differences between different machines. Finally, we cannot draw definitive conclusions regarding other monitors available on the market that differ from BIS and SedLine.

## Conclusion

SEF95 monitored by BIS or SedLine in pediatric patients showed some differences, with deltas up to ± 4 Hz. Values appeared to be closer during the anesthesia maintenance phase. The clinical relevance of these findings should be further confirmed.

## Supplementary Information

Below is the link to the electronic supplementary material.

Supplementary material 1 (DOCX 4276.4 kb)

## Data Availability

Data is provided within the manuscript. Additional or more detailed data may be shared upon reasonable request.
