# Supplementary material for: Analysis of SEF95 derived from two processed EEG devices during pediatric anesthesia for non-cardiac surgery
Source: J Clin Monit Comput. 2025 Aug 13;40(2):317–22. doi: 10.1007/s10877-025-01338-3 (PMC13053526; doi:10.1007/s10877-025-01338-3)

Supplementary figure 1. Sensors placement.


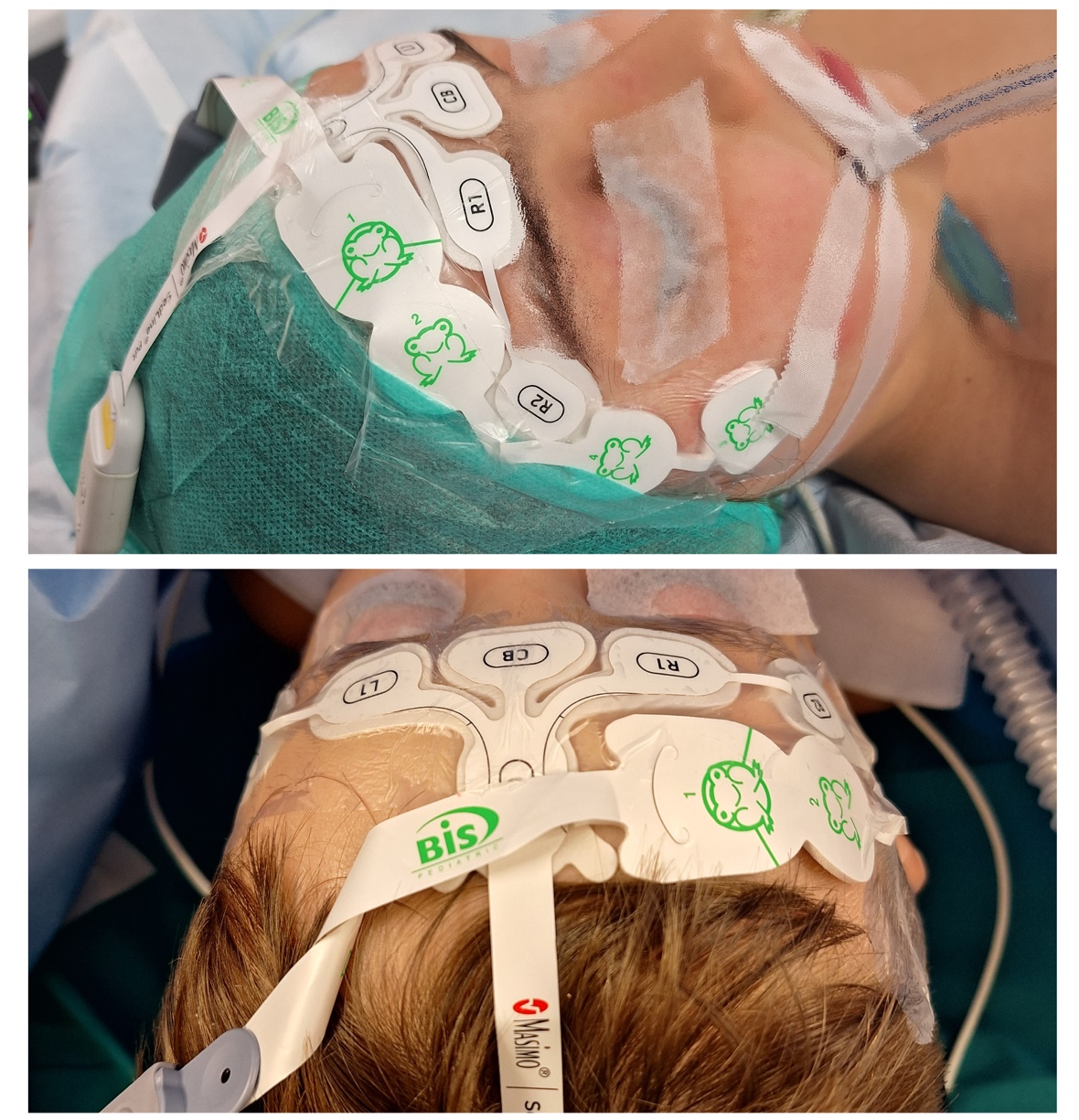


Supplementary figure 2. Enrollment flowchart.


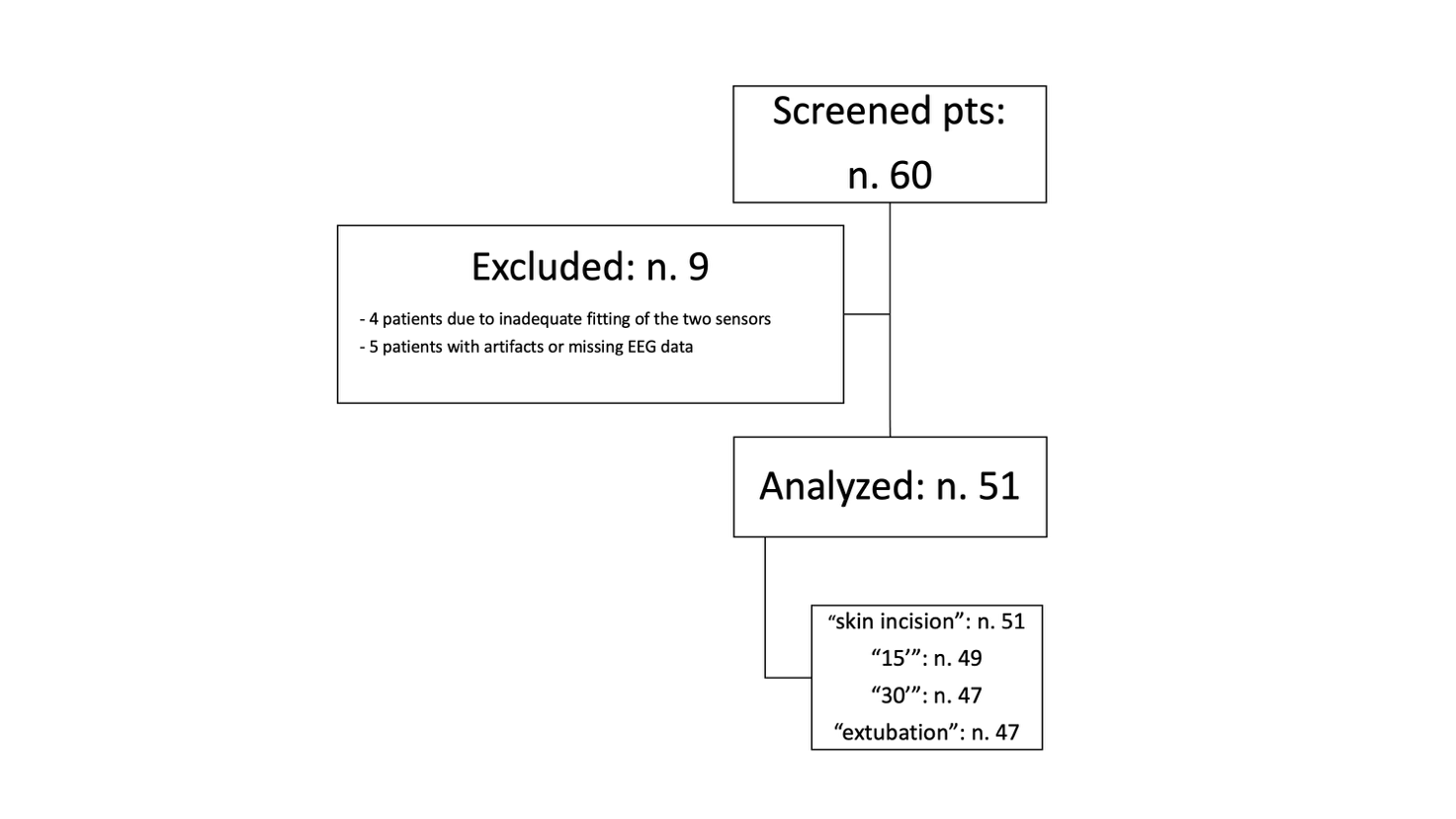


Supplementary figure 3. Heart rate and mean arterial pressure over time.


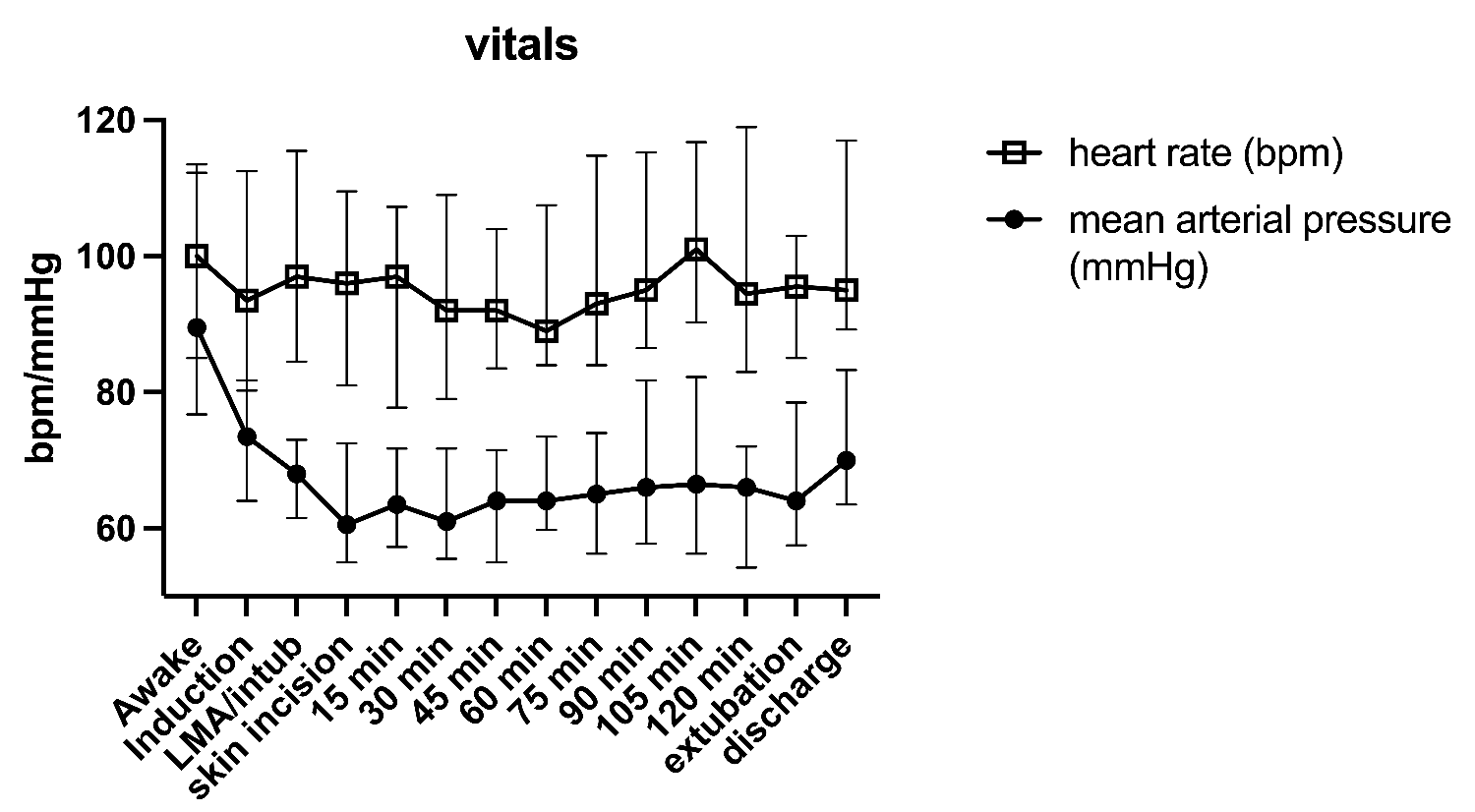

Supplement: Supplementary file 1 — Supplementary material 1 (DOCX 4276.4 kb) [file 10877_2025_1338_MOESM1_ESM.docx]
